# Explainable artificial intelligence for predicting medical students’ performance in comprehensive assessments

**DOI:** 10.1038/s41598-025-07460-1

**Published:** 2025-07-03

**Authors:** Haniye Mastour, Toktam Dehghani, Ehsan Moradi, Saeid Eslami

**Affiliations:** 1 Department of Medical Education, School of Medicine, Mashhad University of Medical Sciences, Mashhad, Iran; 2 School of Medical Education and Learning Technologies, Shahid Beheshti University of Medical Sciences, Tehran, Iran; 3 Department of Medical Informatics, Faculty of Medicine, Mashhad University of Medical Sciences, Mashhad, Iran; 4 Head of Information Technology at Vice President for Education, Mashhad University of Medical Sciences, Mashhad, Iran; 5 Pharmaceutical Sciences Research Center, Institute of Pharmaceutical Technology, Mashhad University of Medical Sciences, Mashhad, Iran; 6 Department of Medical Informatics, UMC-Location AMC, University of Amsterdam, Amsterdam, The Netherlands

**Keywords:** Artificial intelligence, Comprehensive medical assessments, Artificial intelligence in education, Explainable AI, Machine learning, Medical licensing exams, Health care, Health occupations, Medical research, Mathematics and computing

## Abstract

Comprehensive medical assessments are critical for evaluating clinical proficiency in medical education; however, these administrations impose significant institutional burdens, financial costs, and psychological strain on students. While Artificial intelligence (AI) holds transformative potential for predictive analytics, existing models lack the interpretability and reliability required for educational decision-making. To address this gap, a machine learning (ML) framework enhanced with explainable AI (XAI) was developed to predict medical students’ performance on comprehensive assessments by integrating academic metrics and non-academic attributes. This retrospective cohort study validated the framework across three universities using two high-stakes assessments: the Comprehensive Medical Pre-Internship Examination (CMPIE; n = 997 students, two-month prediction horizon) and the Clinical Competence Assessment (CCAs; n = 777 students, one-year horizon). A stacking meta-model that combined ensemble techniques (Random Forest, Adaptive Boosting, XGBoost) demonstrated outstanding discriminative performance, with AUC-ROC values of 0.97 (CMPIEs) and 0.99 (CCAs) as well as F1-scores (0.966, 0.994). In this framework, SHapley Additive exPlanations (SHAP) provided granular insights into model logic by identifying high-impact courses as dominant predictors of success and individualized risk profiles. These insights empower educators to prioritize curriculum reforms and implement early interventions for at-risk students while delivering personalized feedback for learners to enhance learning outcomes.

## Introduction

The quality of medical students’ education is a cornerstone of healthcare systems and directly influences future practitioners’ competency and the efficacy of clinical care delivery ^1^. Medical policymakers globally employ comprehensive assessments as critical benchmarks to evaluate students’ knowledge, clinical skills, and readiness for practice ^2,3^. These assessments, such as the Comprehensive Osteopathic Medical Licensing Examination (COMLEX-USA) in the United States ^4,5^ and Iran’s Comprehensive Medical Pre-Internship Examination (CMPIE) and Clinical Competence Assessment (CCA), determine eligibility for graduation and medical licensure ^6^. However, their administration imposes significant institutional burdens, including financial costs and logistical complexity, while subjecting students to psychological stress ^7^. Researchers increasingly advocate for artificial intelligence (AI)-driven tools to predict exam performance, enabling early identification of at-risk students, personalized academic interventions, and optimized resource allocation ^8^.

The rise of educational analytics and advances in AI and machine learning (ML) have transformed higher education through disciplines like Artificial Intelligence in Education (AIED) and Educational Data Mining (EDM) ^9–11^. These fields leverage predictive modeling to uncover patterns in student behavior, academic performance, and curriculum efficacy, which empower institutions to refine pedagogical strategies, enhance retention, and tailor support to individual needs ^12–16^. Classical ML algorithms (e.g., Logistic Regression (LR) ^17^, K-Nearest Neighbors (KNN) ^18^, Support Vector Machines (SVM) ^19^, Artificial Neural Networks (ANN) ^20^, Decision Trees (DT) ^21^, and Naïve Bayes (NB) ^22^), ensemble methods (e.g., Random Forest (RF) ^23^, Adaptive Boosting (ADA) ^24^, Extreme Gradient Boosting (XGB) ^25^), and meta-learning models (e.g., stacking ^26^), have demonstrated utility in educational performance prediction. However, the adoption of advanced ML in high-stakes medical education remains limited due to inherent "black-box" opacity. Educators often mistrust models that lack interpretability, mainly when these models are utilized in decision-making, which impacts student progression or institutional policy ^27^.

To bridge this gap, explainable AI (XAI) techniques, such as Shapley additive explanations (SHAP), have emerged as critical tools for elucidating model logic ^28–30^. SHAP quantifies attribute contributions to predictions through game-theoretic principles, generating global and instance-level explanations that align with educators’ need for transparency ^29,31,32^. However, few prior studies have utilized XAI in medical education, focusing narrowly on academic predictors while neglecting non-academic factors, the temporal dynamics of sequential assessments (pre-internship to clinical competence phases), and cross-institutional generalizability ^33–39^.

This study proposes an interpretable ML framework to predict performance in sequential, comprehensive medical assessments across three universities. Our models identify at-risk students and curriculum bottlenecks months to a year in advance based on academic metrics and non-academic attributes. The framework employs a stacking meta-model to optimize accuracy and SHAP plots to decode attribute importance at cohort, course, and individual levels. For educators, this enables targeted interventions such as curriculum redesign and proactive counseling. For students, it provides transparent feedback to self-regulate learning.

## Method and materials

**Fig. 1:**
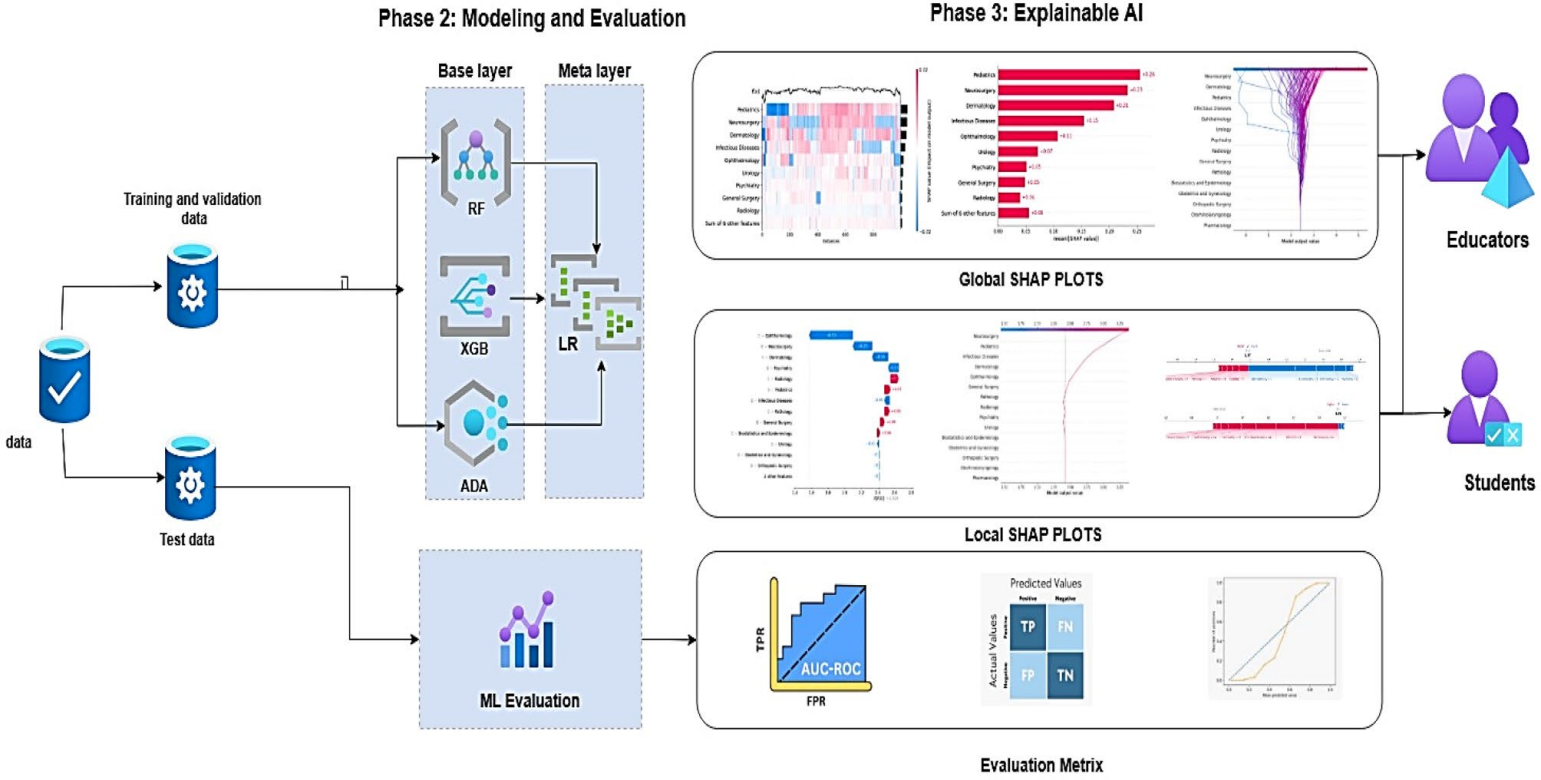
ML for Predicting Medical Students’ Performance in Comprehensive Assessments (CMPIEs and CCAs). Note: An ML framework designed to predict medical students’ performance in Comprehensive assessments (CMPIEs and CCAs) in three phases: (1) Preprocessing phase addresses (cleaning, attribute transformation, and SMOTE resampling), (2) Modeling phase (two-layer stacking meta-model: base models (Random Forest (RF), AdaBoost (ADA), XGBoost(XGB)) are aggregated by a logistic regression (LR) meta-learner), and Evaluation phase (nested cross-validation and hyperparameter tuning). (3) Explainable AI (SHAP plots) for educators and learners.

This paper presents an interpretable ML framework to predict medical student performance in two sequential, comprehensive assessments: the Comprehensive Medical Pre-Internship Examination (CMPIEs) and Clinical Competence Assessments (CCAs). This retrospective cohort study validated the framework across three Iranian medical universities. We analyzed academic and non-academic data from 997 students (2017–2022). The workflow (Fig. 1) and research questions (Table 1) address gaps in predictive accuracy, generalizability, and interpretability for medical education contexts. Informed consent was waived by the Iran National Committee for Ethics in Biomedical Research (NASR). Additionally, we adhere to the Transparent Reporting of a Multivariable Prediction Model for Individual Prediction or Diagnosis (TRIPOD) guidelines ^40^.

**Table 1:** Research questions.

RQ	Research question
RQ1	To what extent do ML models demonstrate reliable predictive performance for predicting student outcomes across medical assessments?
RQ2	How can explainable AI (XAI) techniques systematically identify (a) critical academic/non-academic predictors of success, (b) course-specific bottlenecks impacting cohort-wide performance, and (c) individualized risk profiles to perform actionable pedagogical insights by educators?

### Data collection

**Fig. 2:**
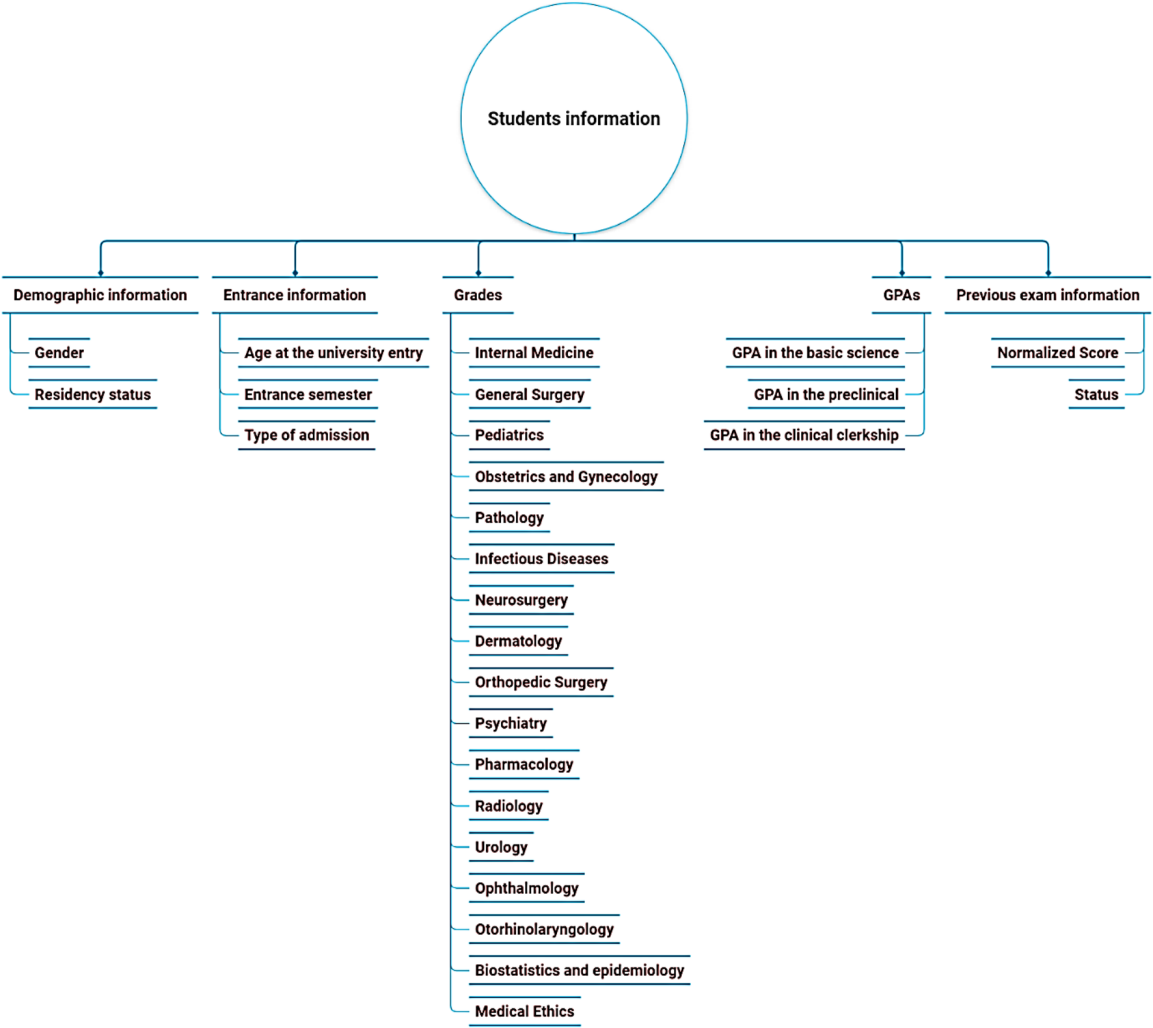
Predictive Attribute for Comprehensive Medical Assessments Outcomes.

Data were extracted from Iran’s Comprehensive Education System (CES), integrating five dimensions (Fig. 2): (1) demographics (gender and residency status), (2) admission metrics (age at entry, entrance semester, and admission type), (3) clinical clerkship grades (16 specialties, e.g., Internal Medicine, Surgery, Pediatrics), (4) phase-specific GPAs (basic sciences, preclinical, clinical), and (5) historical CMPIEs performance (normalized scores, pass/fail status). The CMPIEs, administered in Year 5, assess clinical discipline mastery via written exams, while the CCAs (Year 6) evaluate clinical reasoning through standardized patient encounters.

### Preprocessing of data

This study’s preprocessing step involved significance testing, cleaning, redundancy reduction, and imbalance mitigation. First, a Chi-square test identified attributes with significant differences between pass/fail groups (p < 0.05). Second, missing data (e.g., due to student transfers, withdrawals, or major changes) reduced the cohort to 997 (CMPIEs) and 777 (CCAs). Third, categorical variables were one-hot encoded. Finally, Cramer’s V (> 0.8) was applied to eliminate redundant categorical pairs ^41^.

**Fig. 3:**
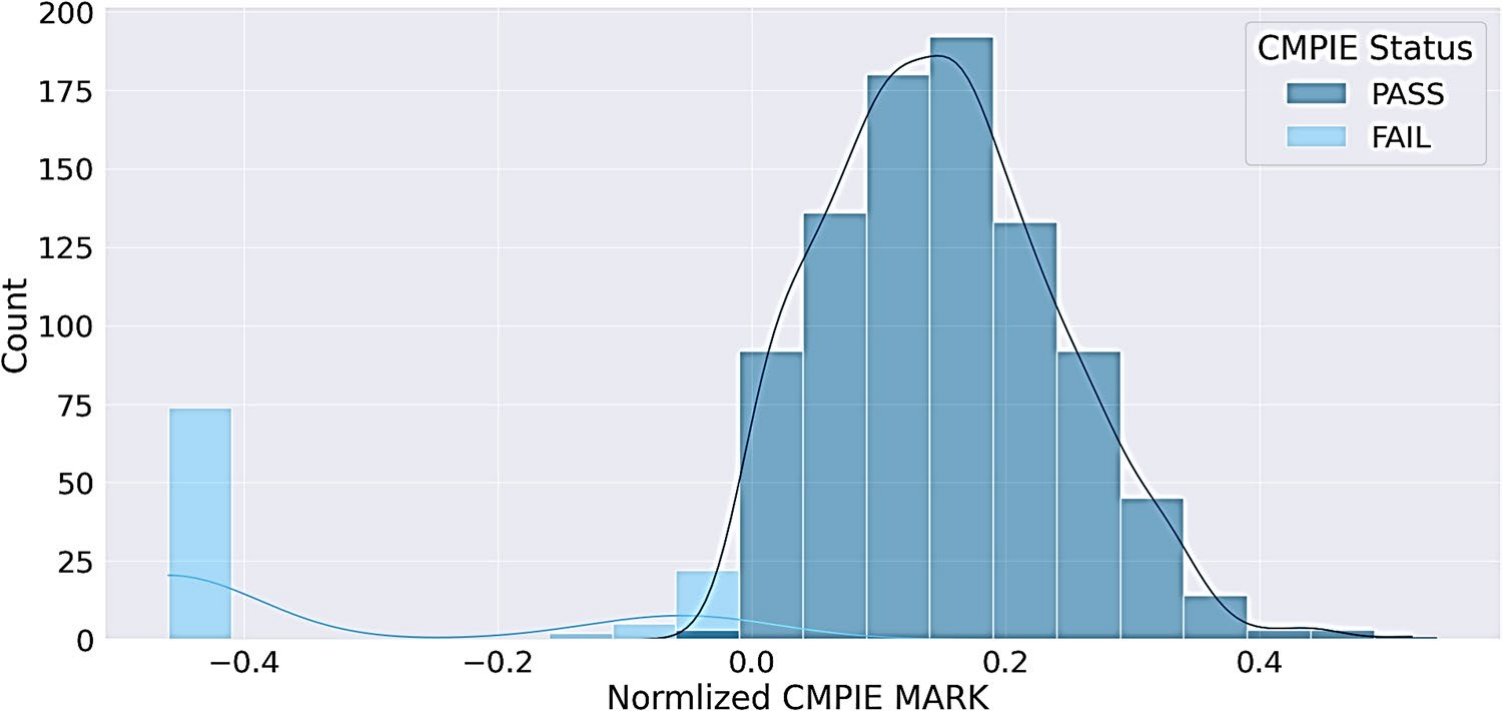
Imbalanced Distribution of Normalized CMPIE Scores. Note: A right-skewed distribution pattern in normalized CMPIE scores, where first-time test takers exhibited a mean score of 0.098 ± 0.183 on the normalization scale.

Severe class imbalance was observed: 90% passed CMPIEs (897 vs. 100 failed), and 95% passed CCAs (738 vs. 39 failed). Also, Further analysis revealed a right-skewed distribution pattern in normalized CMPIE scores (Fig. 3), where first-time test takers exhibited a mean score of 0.098 ± 0.183. To address this, seven resampling techniques: oversampling (ROS, SMOTE, Borderline SMOTE), which artificially increases minority-class examples by generating synthetic instances; undersampling (RUS, Tomek Links, ENN), which removes redundant or noisy majority-class samples; and hybrid (SMOTE-ENN, SMOTE-Tomek) ^42–50^, which combine oversampling with undersampling to optimize class distribution while preserving critical data patterns, were evaluated. The optimal technique, determined via LR performance, was applied to the training dataset of ML models.

### ML models development for predicting medical students’ performance

In this study, a two-phase framework was developed to predict outcomes in sequential comprehensive assessments:

#### Phase 1 (CMPIE outcome prediction):

Three ensemble models—RF, ADA, and XGB—were trained on 26 attributes, including demographics, academic history, and admission details. RF leverages an assembly of decision trees through bootstrap aggregation ^23^. ADA iteratively adjusts weights for misclassified samples via adaptive boosting and emphasizing challenging cases ^51^. XGB enhances gradient-boosted trees with regularization and parallel processing ^25^. The stacking meta-model ^26^ unified their predictions by employing LR as the meta-learner, synthesizing complementary strengths of the base models to enable granular risk stratification 1–2 months pre-exam.

#### Phase 2 (CCA outcome prediction):

A second stacking model incorporated Phase 1 predictions (predicted Status in CMPIE) and the same 26 attributes to predict CCA outcomes one year in advance, capturing temporal dependencies between assessments.

### Evaluating ML models’ performance

Model performance was evaluated using precision, recall (sensitivity), specificity, accuracy, F1-score, and AUC-ROC ^52–56^ (See definitions in Table 2). To rigorously address model generalizability and mitigate overfitting, first, 33% of the dataset was randomly reserved as an independent test set, entirely excluded from model construction and hyperparameter tuning phases. The remaining 67% underwent nested cross-validation (5 outer folds for performance estimation and three inner folds) combined with GridSearchCV ^57^ to optimize hyperparameters while preventing data leakage. Final performance metrics were computed exclusively on the held-out test set, ensuring an unbiased assessment of real-world applicability.

**Table 2:** Evaluation Metrics for evaluation of ML framework.

Metrics	Definition
Precision (PPV)	$$\raisebox{1ex}{$\text{TP}$}\!\left/ \!\raisebox{-1ex}{$\left(\text{TP}+\text{FP}\right)$}\right.$$
Sensitivity (recall)	$$\raisebox{1ex}{$\text{TP}$}\!\left/ \!\raisebox{-1ex}{$\left(\text{TP}+\text{FN}\right)$}\right.$$
Specificity (Spe)	$$\raisebox{1ex}{$\text{TN}$}\!\left/ \!\raisebox{-1ex}{$(\text{TN }+\text{ FP})$}\right.$$
Accuracy (Acc)	$$\raisebox{1ex}{$(\text{TP}+\text{TN})$}\!\left/ \!\raisebox{-1ex}{$(\text{TP}+\text{FP}+\text{FN}+\text{TN})$}\right.$$
F1-measure (F1)	$$\raisebox{1ex}{$2\times (\text{Precision}\times \text{Recall})$}\!\left/ \!\raisebox{-1ex}{$(\text{ Precision}+\text{Recall})$}\right.$$

The variables used in the equation are defined as follows: True Positive (TP) represents the number of students correctly predicted to pass the exam. A False Negative (FN) indicates the number of students wrongly predicted to fail. True Negative (TN) represents the number of students correctly predicted to fail, and False Positive (FP) represents the number of students wrongly predicted to pass the exam.

### Explainable AI for predicting students’ performance

SHapley Additive exPlanations (SHAP) quantified attribute contributions to predictions ^58^. Global interpretation used heatmap, bar, and decision plots to identify cohort-level drivers, while force/waterfall plots provided instance-level explanations for individual students.

## Results

This study utilized Python (Scikit-learn, Pandas, NumPy) within the Google Colab IDE to develop, evaluate, and visualize models. The source code for our work is accessible at the following link: https://github.com/smartdxgen/XAI-Medical-Comprehensive-Assessments/ and https://mega.nz/folder/Tx90HZaJ#H4EuGfp6C5DuDifpEL3ABQ/file/PsFCCBwK.

### Statistical analysis of student attributes

Chi-square tests identified significant differences between students who passed (*n* = 897) and failed (*n* = 100) the CMPIEs. Seventeen of 22 clerkship courses (e.g., Internal Medicine, Surgery) showed divergent performance (*p* < 0.05). Orthopedic Surgery, Pharmacology, Radiology, Otorhinolaryngology, and Biostatistics/Epidemiology exhibited no significant differences (Table 3). Also, demographic variables (gender and admission type) and entrance age showed no significant associations with outcomes, while residency status and entrance semester differed significantly between groups.

**Table 3:** Comparison of attributes between students who passed or failed CMPIEs.

Category	Attribute name	Values	Test statistic	*P*-value
Demographic information	Gender Residency status	{Male, Female} {Native, Non-native}	2.149 7.347	0.143 ** < 0.05**
Entrance/ admission information	Age at the university entry Entrance semester Type of admission	{Below 20, 20, above 20} {Autumn, Spring} {Free, Paying tuition}	2.808 13.751 0.553	0.246 ** < 0.001** 0.457
Grades in clinical clerkship curriculum (CCC)	Internal medicine General surgery Pediatrics Obstetrics and gynecology Pathology Infectious diseases Neurosurgery Dermatology Orthopedic surgery Psychiatry Pharmacology Radiology Urology Ophthalmology Otorhinolaryngology Biostatistics and epidemiology Medical ethics	{F, D, B, A, A +}	15.929 15 9.788 11.187 16.923 18.274 30.019 71.999 5.986 8.731 9.282 3.149 29.086 29.154 4.841 2.259 11.686	** < 0.001** ** < 0.05** ** < 0.05** ** < 0.05** ** < 0.05** ** < 0.001** ** < 0.001** ** < 0.001** 0.31 ** < 0.05** 0.10 0.68 ** < 0.001** ** < 0.001** 0.18 0.69 ** < 0.05**
Grade point average (GPA)	GPA in the basic science phase GPA in the preclinical phase GPA in the clinical clerkship	{F, D, B, A, A +}	22.503 17.267 2.537	** < 0.001** ** < 0.05** 0.281
Exam information	Age at the time of the exam	{Below 25, 25, above 25}	3.262	0.196

* Attributes with statistically significant differences between two groups (*p* < 0.05) are indicated by bolding the corresponding values.

**Fig. 4:**
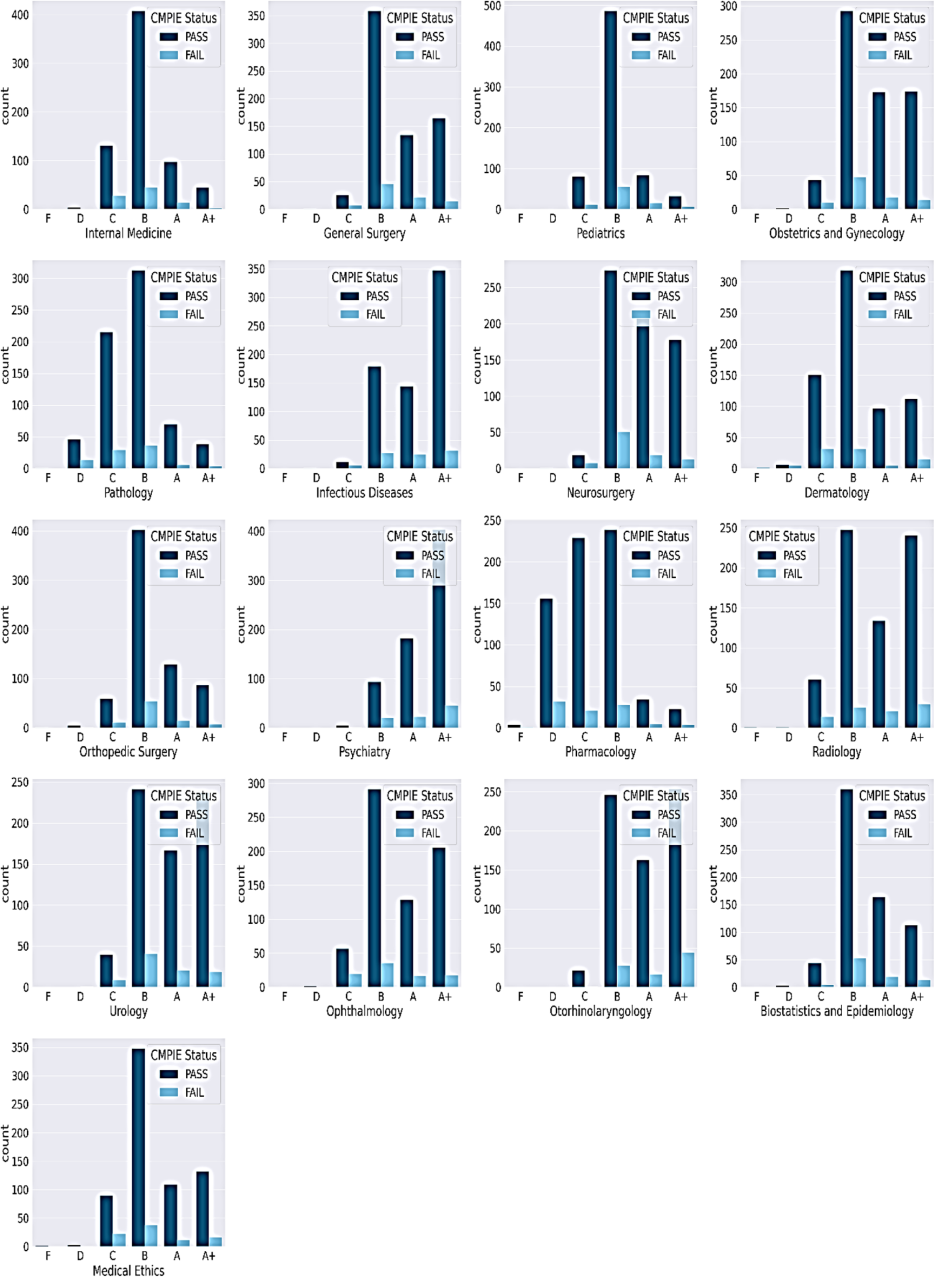
Comparison of Grade Distribution Between Passed or Failed Students in CMPIEs.

**Fig. 5:**
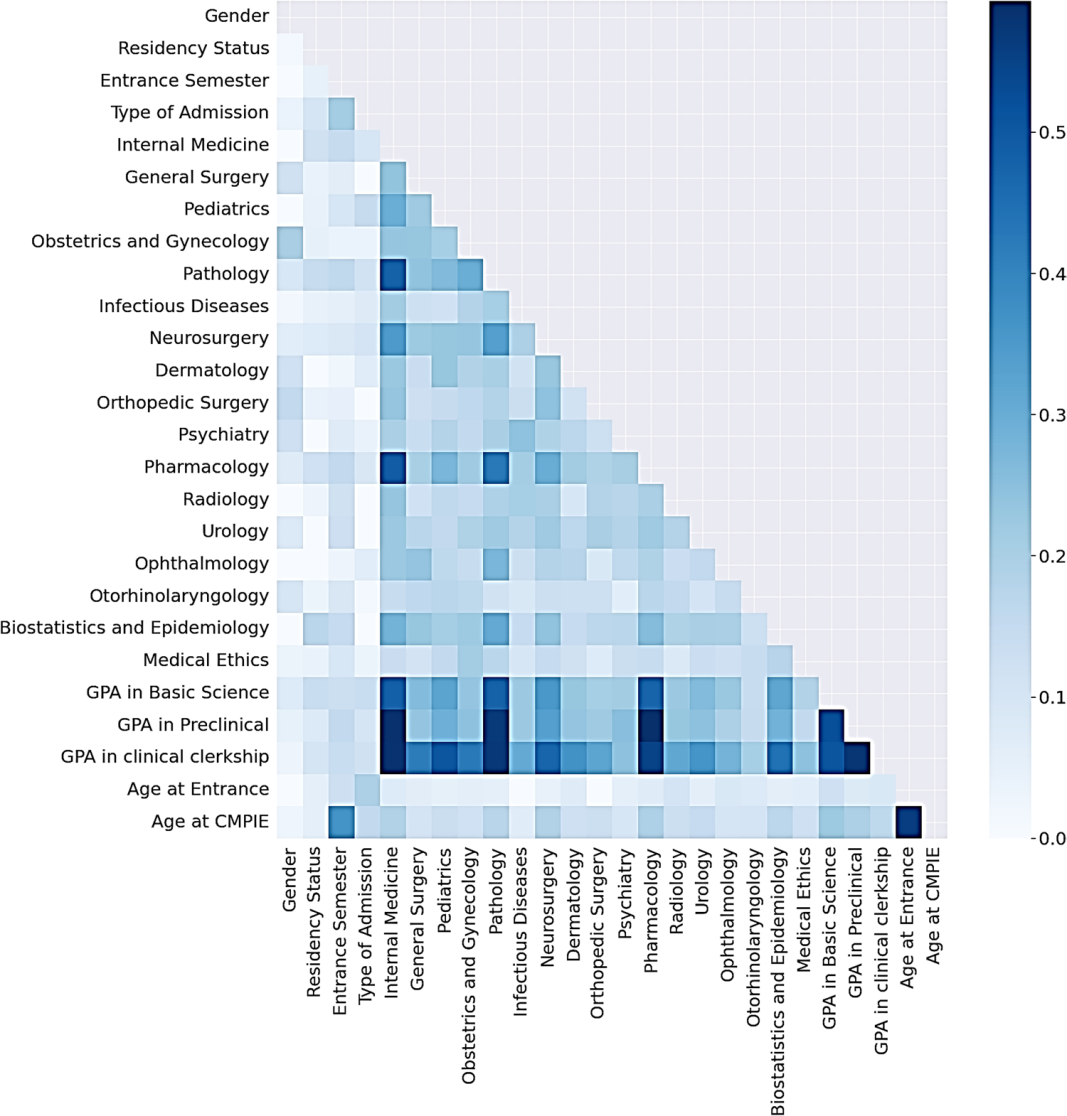
Pairwise Correlations Between Attributes of Passed or Failed Students in CMPIEs. Note: This heatmap visualizes pairwise correlations between student attributes. Darker hues represent stronger correlations (coefficients approaching ± 1), while lighter hues indicate weaker or negligible associations (coefficients near 0).

Grade distributions revealed academic trends that passing students predominantly scored ≥ B in most courses. However, nearly half of passing students earned C/D grades in Pharmacology and Pathology (Fig. 4), suggesting these courses pose systemic challenges even for high performers. Pairwise correlations highlighted moderate associations between preclinical/clinical GPAs and grades in Pharmacology, Pathology, and Internal Medicine (Fig. 5). No attributes exceeded redundancy thresholds (> 0.8); therefore, the predictive breadth was preserved.

### Addressing class imbalance

The dataset exhibited severe class imbalance, with 90% passing CMPIEs and 95% passing CCAs. To mitigate bias, seven resampling strategies were evaluated, with SMOTE-ENN (a hybrid method) achieving optimal class balance (51% passed vs. 49% failed. Performance was validated on a fully independent test set excluded from resampling, hyperparameter tuning, and model training phases. A comparative analysis between the non-augmented baseline model and the augmented baseline model revealed stark contrasts (Table 4): the non-augmented baseline model showed near-random discrimination (AUC = 0.59), with low minority class performance (precision = 0.27, recall = 0.09, F1 = 0.13). After controlled SMOTE-ENN augmentation (capped at oversampling of minority samples), the model achieved significant improvements: minority class precision surged to 0.95, recall to 0.95, and F1 to 0.94, while majority class precision improved to 0.94 with an intentional recall trade-off (0.95 to 0.89). The 33 percentage-point AUC-ROC gain (0.59 to 0.92) and stable accuracy (0.92) were corroborated by the independent test set, confirming that synthetic data enhanced discriminative learning.

**Table 4:** Logistic Regression Performance Before/After SMOTE-ENN Resampling.

Metric	Class	Before resampling	After resampling
Precision	Minority (fail)	0.27	0.95 ↑
	Majority (pass)	0.90	0.94 ↑
Recall	Minority (fail)	0.09	0.95 ↑
	Majority (pass)	0.97	0.89 ↓
F1-Score	Minority (fail)	0.13	0.95 ↑
	Majority (pass)	0.94	0.92 ↓
AUC-ROC	Overall	0.59	0.92 ↑

### Evaluation of ML models for comprehensive medical assessments

To evaluate the efficacy of the proposed framework in predicting student outcomes on the CMPIE and CCA, we conducted comparative assessments: (1) baseline models (LR), (2) individual ensemble methods (RF, ADA, XGB), and (3) our stacking meta-model (RF, ADA, XGB + LR meta-learner).

#### CMPIEs prediction performance

For CMPIE prediction, while simpler baseline models (LR) achieved reasonable performance (AUC 0.82–0.85), the stacked approach provided statistically significant improvements (AUC 0.97, p < 0.01). Notably, while RF showed marginally better AUC (0.98 vs 0.97), the stacked model’s consistent excellence across all evaluation dimensions, accuracy (0.98, 95% CI: 0.96–1.00), precision (0.97, 95% CI: 0.94–0.99), recall (0.96, 95% CI: 0.93–0.98), F1-score (0.97, 95% CI: 0.95–0.99), and AUC-ROC (0.97, 95% CI: 0.95–0.99) (Table 5).

**Table 5:** Comparative analysis of ML models for predicting CMPIE outcomes.

ML model	Accuracy	Precision	Recall	F1	AUC
LR	0.81	0.85	0.72	0.78	**0.82**
RF	0.92	**0.97**	0.82	0.89	**0.98**
ADA	0.84	0.94	0.61	0.74	0.94
XGB	0.96	0.95	0.96	0.95	0.96
Stacked	**0.98**↑	**0.97**↑	**0.96**↑	**0.97**↑	**0.97**↓

* The highest value in each metrics is indicated by bolding the corresponding values.

**Fig. 6:**
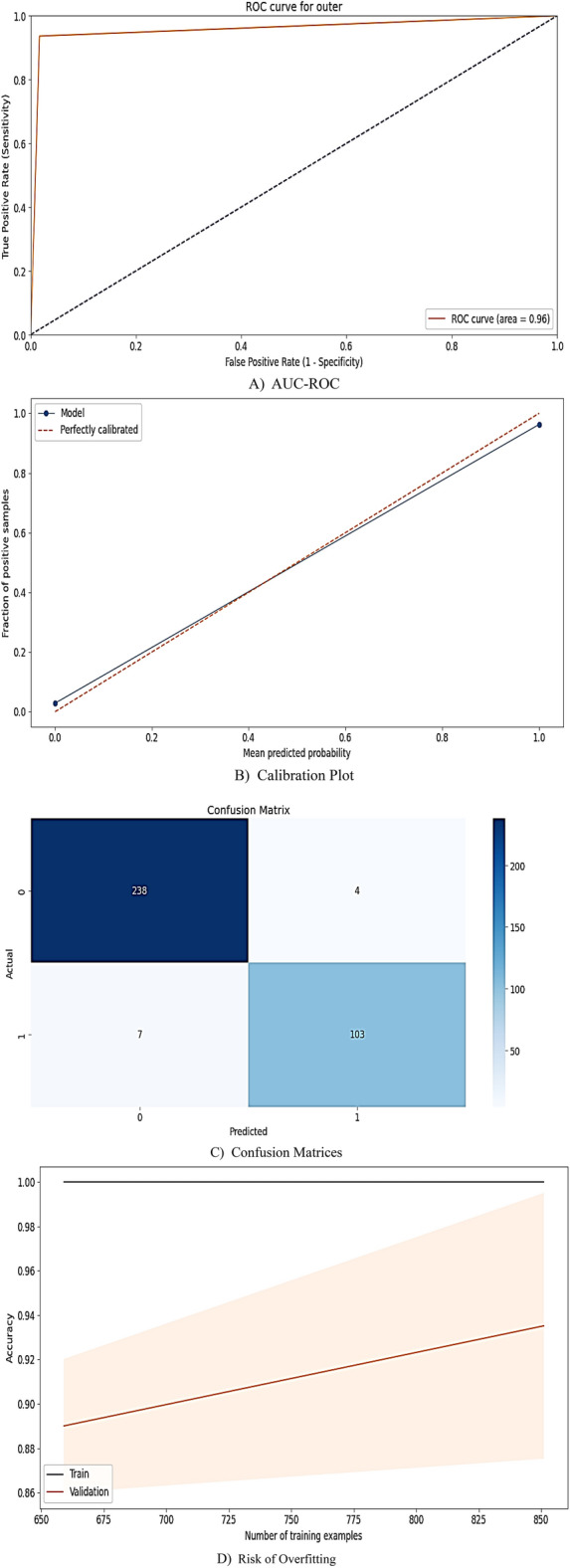
Four-Dimensional Reliability Assessment of Stacking Model for Prediction of CMPIEs Outcome. Note: (**A**) Discriminatory Power (AUC-ROC), (**B**) Class-Specific Calibration, (**C**) Prediction-Observation Consistency (Confusion Matrix), (**D**) Overfitting Analysis (Training vs. Test Performance).

The reliability of the stacking model was comprehensively assessed across four critical dimensions (Fig. 6). Evaluated via AUC-ROC, the discriminatory power demonstrated high performance with an overall value of 0.97, reflecting robust separation between passed and failed students (Fig. 6A). Calibration analysis (Fig. 6B) revealed a near-perfect alignment between predicted probabilities and observed outcomes. The calibration curves indicated for minority (fail) and majority (pass) classes closely follow the ideal diagonal. Disparities between pass/fail classes in precision (Δ = 0.013), recall (Δ = 0.022), and F1-score (Δ = 0.004) were negligible, which indicates the model’s fairness in high-stakes settings.

Prediction consistency was validated through confusion matrices (Fig. 6C), which showed high true positive (TP = 98%) and true negative (TN = 97%) rates. Finally, the overfitting risk of the ML model was mitigated by comparing performance on training and test datasets (Fig. 6D). The marginal divergence between the training and testing phases, as indicated by the AUC (Δ < 0.04) and accuracy (Δ < 0.09), confirms robust generalizability.

#### CCAs prediction performance

**Fig. 7:**
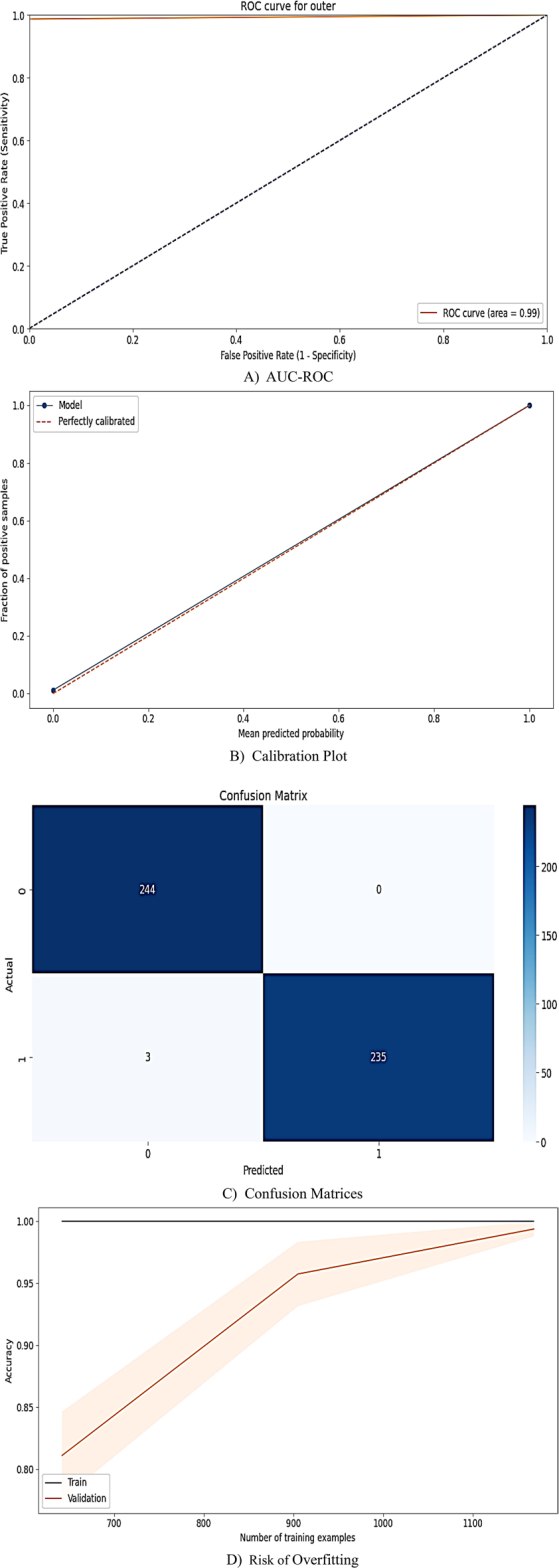
Four-Dimensional Reliability Assessment of Stacking Model for Prediction of CCAs Outcome. Note: (**A**) Discriminatory Power (AUC-ROC), (**B**) Class-Specific Calibration, (**C**) Prediction-Observation Consistency (Confusion Matrix), (**D**) Overfitting Analysis (Training vs. Test Performance).

As Fig. 7A indicates, the stacking meta-model achieved near-perfect performance (AUC-ROC = 0.99, precision = 0.993, recall = 0.987, F1-score = 0.994). Calibration curves showed minimal deviation (< 0.02) from ideal predictions (Fig. 7B). The confusion matrices confirmed zero false negatives (FN) and a 99.1% true negative (TN) rate (Fig. 7C). The training-test analysis demonstrated robust generalizability (test accuracy: 0.80 to 0.99; Fig. 7D).

### Explainable ML models for comprehensive medical assessments

This study employed SHAP plots to elucidate the global and individualized contributions to enhance the predictive framework’s trustworthiness and applicability.

#### Global interpretability

**Fig. 8:**
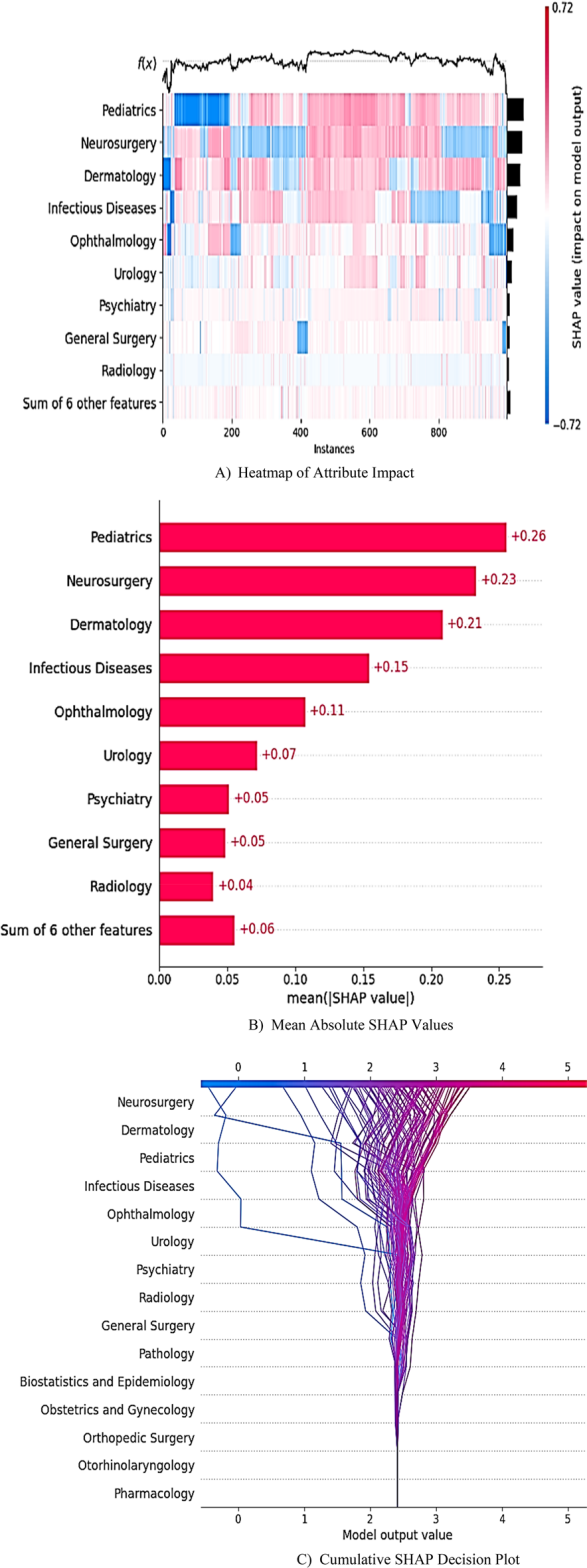
SHAP Analysis of Important Predictors for CMPIE Performance. Note: (**A**) Heatmap Plot: Red hues denote higher grades, and blue hues indicate lower grades. Each column represents a student instance; (**B**) Bar Plot: Bar length corresponds to the mean absolute SHAP value, quantifying overall attribute importance. (**C**) Decision Plot: Each line represents a learner’s cumulative SHAP value progression, with the terminal position relative to the passing threshold determining their predicted outcome (blue = fail, red = pass).

The integration of global SHAP plots, including heatmaps, bar plots, and cumulative decision plots (Fig. 8), provided a comprehensive understanding of model logic across 977 student records. The SHAP heatmap (Fig. 8A) offers a detailed visual summary of attribute impacts, where columns represent individual students and rows correspond to course grades ranked by SHAP values (indicating their impact on the model’s outcomes). Red hues indicate positive SHAP values and signify increases in pass probability linked to higher grades. Blue hues represent negative values and highlight elevated risks of failure. The vertical pattern in the plot revealed the attributes’ importance, with Pediatrics, Neurosurgery, and Dermatology emerging as dominant predictors. Horizontal patterns illustrated how high grades (red clusters) in these disciplines correlated strongly with passing outcomes (f(x) > 2.4), and at-risk students (blue clusters) exhibited persistent deficiencies.

Bar plots (Fig. 8B) quantified these trends by showing Pediatrics (mean |SHAP|= 0.26), Neurosurgery (0.23), and Dermatology (0.21) as the most influential attributes, which collectively explain 70% of predictive power. Infectious Diseases (0.15) and Ophthalmology (0.11) contributed secondarily, and six low-impact courses (e.g., Urology, Psychiatry) accounted for < 5% of model decisions. Students with ≥ B grades in top-tier disciplines demonstrated significantly higher pass probabilities (*p* < 0.001).

The cumulative SHAP decision plot (Fig. 8C) traced the trajectory of attribute contributions for 100 representative students. Red trajectories (terminal f(x) > 2.4) reflected passing outcomes driven by high Neurosurgery, Pediatrics, or Dermatology grades. In contrast, blue trajectories (f(x) < 2.4) highlighted at-risk students with deficiencies in these critical courses. This visualization confirmed that most passing students achieved A/B grades in at least two high-impact disciplines, whereas failing students consistently underperformed across all key domains.

#### Individualized explanations

Local SHAP analysis provided granular insights into personalized risk profiles. Two contrasting scenarios illustrate this capability:

### Scenario 1 (at-risk student)

**Fig. 9:**
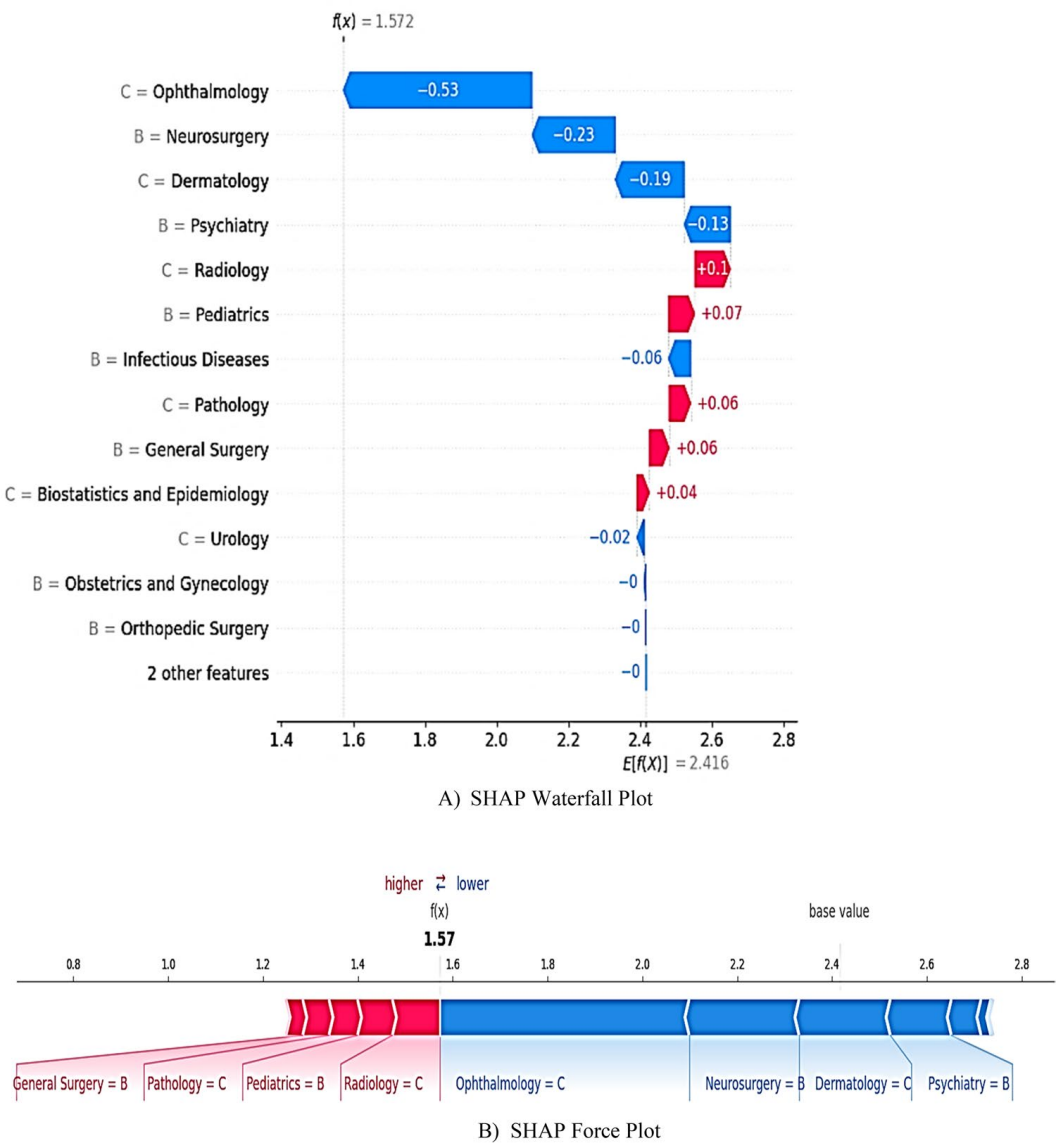
Local SHAP Analysis for an At-Risk Student. Note: Color gradients encode SHAP values, quantifying each attribute’s contribution by fairly allocating the difference between a learner’s prediction and the cohort average. Red hues (positive SHAP values) indicate grade-driven increases in pass probability, whereas blue hues (negative values) signal elevated failure risk. The passing threshold is the expected value E(f(x)) = 2.416. For this student, the predicted outcome f(x) = 1.57, indicating a strong likelihood of failure.

For a student predicted to fail (Fig. 9A), a waterfall plot attributed failure risk (final score = 1.57) to low Ophthalmology (SHAP = -0.53) and Dermatology (-0.19) grades, compounded by moderate Neurosurgery (-0.23) performance. Despite adequate Radiology and Pediatrics scores, deficiencies in high-impact courses drove the prediction below the threshold. Also, the force plot (Fig. 9B) reveals that a C in Ophthalmology, a B in Neurosurgery, a C in Dermatology, and a B in Psychiatry drove the prediction toward failure despite adequate performance in Radiology, Pediatrics, Pathology, and General Surgery.

### Scenario 2 (high-performing student)

**Fig. 10:**
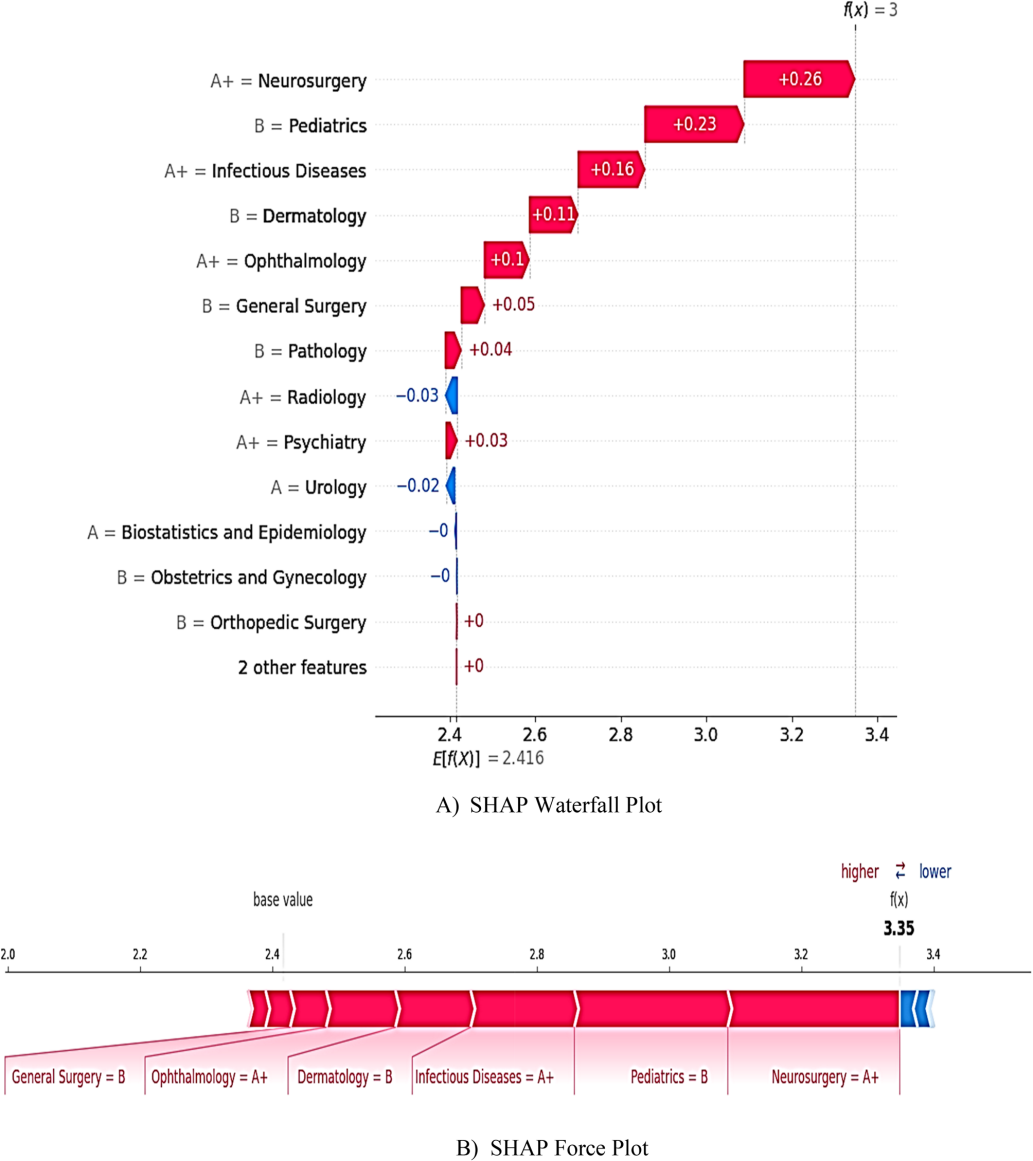
Local SHAP Analysis for a High-Performing Student. Note: Color gradients encode SHAP values, quantifying each attribute’s contribution by fairly allocating the difference between a learner’s prediction and the cohort average. Red hues (positive SHAP values) indicate grade-driven increases in pass probability, whereas blue hues (negative values) signal elevated failure risk. The passing threshold is the expected value E(f(x)) = 2.416. For this student, the predicted outcome f(x) = 3.35, indicating a strong likelihood of passing.

The analysis of a passing student (Fig. 10A) demonstrated that dominant positive contributions from Neurosurgery (SHAP =  + 0.26), Pediatrics (+ 0.23), and Infectious Diseases (+ 0.16) elevated the score to 3.35. Additionally, the force plot (Fig. 10B) showed that A + grades in Neurosurgery and Infectious Diseases offset weaker performance in non-critical courses, such as Radiology and Urology.

## Discussion

Early research demonstrated the utility of academic metrics like GPA in predicting high-stakes exam performance ^1,59–61^. However, most of these studies’ reliance on single-institution cohorts and linear assumptions limited actionable insights. Modern ML frameworks ^34,36–39^ have improved accuracy through classical and ensemble architectures. For instance, Qahmash et al. ^35^ reported that ANN outperformed DT, RF, and NB classifiers (accuracy: 0.743). Mastour et al. ^33^ achieved AUC-ROC and accuracy values of 0.813 and 0.803 with RF models for the Comprehensive Medical Basic Sciences Examination (CMBSE). Narrow input attributes, interpretability, lack of multi-institutional validation, and generalizability continue to be limitations of current methods.

In this study, by combining academic and non-academic data from three universities, our interpretable stacking meta-model overcomes these constraints and achieves high predictive accuracy (Accuracy: 0.98; AUC-ROC: 0.97–0.99; F1-scores: 0.95–0.994). With accuracy gains (> 0.12), this performance outperforms previous models ^33,35^. Also, four major innovations drive this progress: First, the framework integrates multi-dimensional inputs, comprising 26 academic and five non-academic attributes. Second, SMOTE-ENN resampling enhances AUC-ROC by 33% and reduces significant class imbalance (90–95% pass rates). Third, the sequential dependencies between CMPIE and CCA results are captured by temporal integration. Finally, the framework facilitates early risk stratification by identifying at-risk students up to a year before assessments.

This XAI framework transforms predictive analytics into actionable pedagogical strategies through three key mechanisms. First, global SHAP analysis identifies Pediatrics, Neurosurgery, and Dermatology as dominant predictors (70% collective influence), enabling evidence-based curriculum prioritization, such as redesigning Pharmacology modules where systemic bottlenecks persist (e.g., 50% of passing students achieved grades of C or lower). Second, individualized SHAP profiles decode student-specific risks and strengths (e.g., deficiency in Ophthalmology (SHAP =  − 0.53) triggers targeted remediation, while excellence in Neurosurgery (SHAP =  + 0.26) guides peer-mentoring opportunities). Third, the empirically validated decision threshold (f(x) = 2.416) stratifies risk, flagging students scoring below 2.4 for mandatory counseling and those above 3.0 as peer mentors.

This framework empowers educators to (1) allocate resources to high-impact disciplines, (2) set competency benchmarks (≥ B grades in courses with SHAP > 0.20), and (3) deliver personalized feedback (e.g., students with borderline grades in Dermatology are prompted to pursue pre-exam remediation). By identifying at-risk students up to a year in advance, it further supports proactive academic interventions and equitable resource distribution.

## Limitations and future directions

While our predictive framework demonstrates technical effectiveness, several critical limitations warrant consideration. First, there exists a risk of educators developing over-reliance on XAI, potentially leading to automation bias—the tendency to favor algorithmic suggestions even when they conflict with other evidence. Such uncritical acceptance of XAI outputs without rigorous examination of underlying data assumptions could result in suboptimal or unfair educational decisions. Second, while SHAP plots effectively illustrate feature importance, they have inherent constraints: (1) they may oversimplify complex feature-performance relationships, and (2) they explain model predictions without establishing causal mechanisms. This limitation may hinder educators’ ability to develop targeted curriculum modifications or interventions based solely on these explanations. Third, the implementation of AI for performance prediction raises substantial ethical concerns regarding fairness, privacy, and potential discrimination. Despite XAI’s transparency benefits, ensuring complete model neutrality and equitable application remains challenging. These ethical considerations demand careful scrutiny before deploying XAI systems in medical education contexts.

Future studies should address several key limitations to advance this work. First, incorporating non-academic factors, such as psychosocial stressors, motivations, learning styles, and socioeconomic backgrounds, would enhance the model’s comprehensiveness. Second, while our retrospective validation across three universities shows promise, prospective real-time evaluation and external validation across diverse geographic settings are needed to establish generalizability. Third, real-world implementation studies should examine impacts on critical outcomes, including pass rates, student well-being, and institutional resource utilization. Fourth, integrating deep learning architectures with larger datasets could improve predictive performance. Finally, faculty surveys assessing SHAP’s interpretability in actual educational workflows would help bridge the gap between technical outputs and pedagogical decision-making.

## Conclusions

This study introduces an interpretable ML framework to predict medical students’ performance in sequential, comprehensive assessments and address critical gaps in reliability, interpretability, and actionable insight generation. The proposed stacking meta-model demonstrated high predictive performance by achieving AUC-ROC values of 0.97–0.99 and F1-scores of 0.95–0.994. Predictive robustness was achieved by integrating academic and non-academic attributes and considering dependencies between pre-internship (CMPIE) and clinical competence (CCA) assessments. Also, SMOTE-ENN resampling was utilized to deal with the severe class imbalance and improved AUC-ROC by 33%. These developments allow educators to take proactive measures by ensuring accurate risk stratification months to a year in advance.

Explainable AI techniques (SHAP) analysis elucidated actionable insights at both cohort and individual levels. Pediatrics (mean |SHAP|= 0.26), Neurosurgery (0.23), and Dermatology (0.21) were found to be the most significant academic predictors of success on a global scale. Educators could tailor remediation strategies by using local SHAP visualizations to decode student-specific weaknesses, such as deficiencies in ophthalmology.

This framework bridges predictive analytics and actionable pedagogy for educators, supporting early interventions for at-risk students and curriculum redesign in high-impact disciplines. Institutions acquire a tool to minimize redundant assessments and maximize resource allocation. Furthermore, incorporating psychosocial and socioeconomic factors into future research should broaden the model’s application, and external validation across various institutional and geographic cohorts will improve its generalizability.

## Data Availability

The source code for our work is accessible at the following link: https://github.com/smartdxgen/XAI-Medical-Comprehensive-Assessments/  and https://mega.nz/folder/Tx90HZaJ#H4EuGfp6C5DuDifpEL3ABQ/file/PsFCCBwK.

## Abbreviations

AI: Artificial intelligence
ML: Machine learning
AIED: Artificial intelligence in education
EDM: Educational data mining
XAI: Explainable artificial intelligence
SHAP: Shapley additive explanations
LLMs: Large language models
COMLEX-USA: Comprehensive osteopathic medical licensing examination of the United States
USMLE: The United States medical licensing exam
CMPIE: The comprehensive medical pre-internship examinations
CCA: Clinical competence assessments
MCAT: Medical college admission test
DL: Deep learning
LR: Logistic regression
KNN: K-nearest neighbors
SVM: Support vector machine
ANN: Artificial neural network
DT: Decision tree
NB: Naïve Bayes
RF: Random forest
ADA: ADaptive boosting
XGB: Extreme gradient boosting
Spe: Specificity
Acc: Accuracy
F1: F1-measure
AUC-ROC: Area under curve of receiver operator characteristic
ROS: Random over-sampling
SMOTE: Synthetic minority oversampling technique
RUS: Random under-sampling
